# The effect of polymorphism on polymer properties: crystal structure, stability and polymerization of the short-chain bio-based nylon 52 monomer 1,5-pentanedi­amine oxalate

**DOI:** 10.1107/S2052252522010442

**Published:** 2023-01-01

**Authors:** Zihan Li, Shushu Li, Pengpeng Yang, Xincao Fang, Qingshi Wen, Ming Li, Wei Zhuang, Jinglan Wu, Hanjie Ying

**Affiliations:** a National Engineering Technique Research Center for Biotechnology, State Key Laboratory of Materials-Oriented Chemical Engineering, Jiangsu Synergetic Innovation Center for Advanced Bio-Manufacture, College of Biotechnology and Pharmaceutical Engineering, Nanjing Tech University, No. 30, Puzhu South Road, Nanjing 211816, People’s Republic of China; b Industrial Biotechnology Institute of Jiangsu Industrial Technology Research Institute, Nanjing 211816, People’s Republic of China; University of Iowa, USA

**Keywords:** 1,5-pentanedi­amine oxalate, bio-based nylon 52 monomer, phase transformations, nylon polymerizations

## Abstract

The compound 1,5-pentanedi­amine (PDA) is prepared by biological methods using biomass as raw material. The salt of 1,5-pentanedi­amine oxalate (PDA-OXA) was used directly as the monomer for the preparation of a new bio-based nylon 52 material. High-performance polymer materials require initial high-quality monomers, and crystallization is an essential approach to preparing such a monomer. In this work, three crystal forms of PDA-OXA, the anhydrate, dihydrate and trihydrate, were found and the single crystals of two hydrates were obtained. Their crystal structures were determined using single-crystal and powder X-ray diffraction. The thermal behaviors were characterized by thermodynamic analysis, and the lattice energy was calculated to further explore the relationship between the thermal stability and crystal structure. Detailed computational calculations, Hirshfeld analyses and lattice energy calculations were performed to quantify both the contribution of intra- and intermolecular interactions to the supramolecular assembly, as well as the influence on the stability of the structure. The structure–property relationship between the PDA-OXA crystal forms was established. Moreover, the phase transformation mechanism between the crystalline forms of PDA-OXA has been established, and the control strategy of specific crystal forms was developed from the water activity–temperature phase diagram and relevant thermodynamic data. Finally, the influence of the polymorphism of the monomer and the polymerization methods on the properties of the polymer was investigated. The nylon 52 product obtained showed good appearance, high hardness and thermal stability, the polymer made using the anhydrate as the monomer has better thermodynamic properties than that prepared from the dihydrate, indicating practical industrial application prospects.

## Abstract 2

The structure–property relationship between the 1,5-pentanedi­amine oxalate anhydrate and dihydrate has been analyzed and established. The influence of the polymorphism of the monomer and the polymerization methods on the properties of the polymer was also investigated.

## 1. Introduction

Nylon, scientifically known as polyamide (PA), is an industrial polymer containing recurring units –[NH—CO]– of di­amines and di­carb­oxy­lic acids along the molecular backbone (Lee *et al.*, 2020 ▸). Given its excellent tensile properties, bending properties, compressive strength and other mechanical properties; excellent heat resistance, stable chemical properties and excellent electrical insulation properties (Li, 2019 ▸; Deshmukh *et al.*, 2016 ▸; Kim *et al.*, 2012 ▸), nylon has become an indispensable and important chemical fiber. With the need for environmental protection and the advancement of science and technology, increasing requirements have been placed on the performance of nylon materials, especially the market demand for high-temperature resistant nylon with superb performance (Liu *et al.*, 2019 ▸). In the field of high-temperature nylon, the existing production technology is still far below the international level, and the domestic market is monopolized by multinational giants, mainly relying on imports. Moreover, the extraction and production of their raw materials involve related toxic chemical substances, which have caused serious damage and pollution to the environment (Firdaus & Meier, 2013 ▸; Hashimoto *et al.*, 2011 ▸). Therefore, it is of great practical significance to develop bio-based high-temperature nylon centered on renewable resources such as crops and other biomass resources. New bio-based nylons have become a research hotspot, leading the direction of future material development in the nylon industry owing to their consistency with the national sustainable development concept (Osswald & García-Rodríguez, 2011 ▸, Yang *et al.*, 2019 ▸).

**Figure 1:**
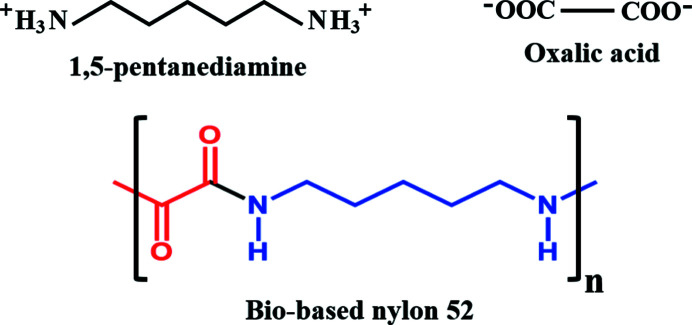
Molecular componentd of 1,5-pentanedi­amine oxalate (top) and the bio-based nylon 52 (bottom).

The bio-nylon series based on 1,5-pentanedi­amine (PDA) is opening up a green route for nylon production owing to its good industry applications (Ogunniyi, 2006 ▸; Hong *et al.*, 2004 ▸). In recent years, a breakthrough has been made in domestic technology for preparing PDA from biological resources (Mi *et al.*, 2021 ▸); thus, the production technology of the nylon 5X series has been established. Nylon 52, which is a polymer formed of the crystalline salts of 1,5-pentanedi­amine-oxalic acid (hereinafter referred to as PDA-OXA, see Fig. 1 ▸, C_7_H_16_N_2_O_4_, MW 192.21 g mol^−1^), has the benefits of a high melting point, low water absorption, acid and alkali resistance *etc.* It is known that high-quality monomers are essential to synthesize excellent-performance polymers, and here crystallization was the main method for the preparation and purification of the monomers (Bosch, 2011 ▸; Burke *et al.*, 2012 ▸). During our crystallization research to improve monomer quality, we found that the PDA-OXA exists in three crystal forms: an anhydrate, a dihydrate and a trihydrate. Unfortunately, despite countless attempts, we could not obtain the single-crystal structure of the anhydrate, nor could we prepare solid powders of the trihydrate after accidentally producing its single crystal.

This phenomenon has aroused our interest: why is the single crystal of the anhydrate so difficult to obtain and why is it so hard to prepare the crystalline products of the trihydrate? Which is the most stable crystalline form suitable for production and storage as a polymerized monomer? These questions prompted us to carry out a series of studies to investigate the crystal structures and physicochemical properties of the three forms. Usually, stability is estimated by thermal analysis or lattice energy calculations to gain additional insight into the target compounds (Zhu *et al.*, 2017 ▸, 2018 ▸). However, intermolecular interactions and packing arrangements also play important roles in predicting crystal forms and their properties (Vener *et al.*, 2014 ▸). Yet there is still a lack of general understanding of crystal structure–stability relationships. Likewise, physicochemical properties such as density, hygroscopicity, solubility, stability and mechanical properties (Cui *et al.*, 2012 ▸; Brittain, 1999 ▸; Fujii *et al.*, 2013 ▸; Liu *et al.*, 2014 ▸, 2018 ▸; SeethaLekshmi *et al.*, 2020 ▸) of the hydrates of a compound may behave differently to the parent anhydrate (Li *et al.*, 2021 ▸). Hence, investigating the transformation mechanism between hydrates and anhydrates can contribute to guaranteeing the stability and quality of the crystalline product (Du *et al.*, 2014 ▸; Steendam *et al.*, 2019 ▸).

In our previous research, we found that there are two important factors – temperature and water activity (*a*_w_), in the experimental system that often determine the transition between hydrate and anhydrate (Li, Yang *et al.*, 2020 ▸; Li, Xu *et al.*, 2020 ▸). Thus, a series of phase transformation experiments, solid-state transformations (SSTs) (Boeckmann & Näther, 2010 ▸; Souza *et al.*, 2002 ▸; Liu *et al.*, 2007 ▸; Zou *et al.*, 2017 ▸) and solution-mediated polymorphic transformations (SMPTs) (Maher *et al.*, 2012 ▸, 2014 ▸; Chee-wei & Ronald, 2006 ▸; Maruyama, 1999 ▸) were designed and conducted to evaluate how these two factors affect the transformation of the crystal forms. Furthermore, we must also keep in mind that the critical values of transformation zones need to be determined. Slurry experiments were a more common method in the SMPT category, which is convenient to assess the relative stability of crystalline products.

This work aims to unravel the transformation mechanism behind hydrate and anhydrate and their relative stabilities by experimental and computational methods. The three crystalline phases of the PDA-OXA were determined from their structures, morphology and thermal behaviors by various analytical and spectroscopic techniques. A detailed analysis of the intermolecular interactions responsible for the packing and stability has been achieved using Hirshfeld surface analysis (HSA) and energy calculations. Besides, the transformation mechanism between the two crystalline powders obtained under different temperatures and solvent conditions was also studied. Finally, the nylon 52 polymerizations were carried out using the anhydrate and dihydrate obtained from PDA-OXA as the raw material monomer, in order to inspect the influence of the polymorphism of the monomer and the polymerization methods on the properties of the polymer. Thermodynamic properties of the nylon products obtained have been further characterized and analyzed.

## 2. Experimental

### 2.1. Materials

1,5-pentanedi­amine (PDA, 99.5% purity, m.p. 180°C, m.w. 102.18 g mol^−1^) was prepared by the whole-cell catalysis method of l-lysine de­carboxyl­ation in our laboratory. Oxalic acid (OXA, 99.5% purity, m.p. 102°C, m.w. 90.03 g mol^−1^) was purchased from Shanghai Macklin Biochemical Co. Ltd. Analytical-grade ethanol (EtOH, 99.5% purity, BP 78.3°C, MW 46.07 g mol^−1^) was obtained from Shanghai Chemistry Reagent Co. (China). Deionized water was obtained from an ultrapure water system (YPYD Co. China).

### 2.2. Preparation of the crystal forms

#### 2.2.1. Anhydrate

The anhydrate of PDA-OXA was prepared by reaction crystallization in ethanol. Approximately 3.6 ± 0.1 g of PDA was weighed and dissolved in 50 ml of ethanol, then 100 ml of the ethanol solution was poured into a 250 ml double-jacketed crystallizer, and the temperature was kept at 40°C (controlled by a temperature circulation pump, CK-4005GD, SCIENTZ). Then 3.17 g of OXA crystalline powder was slowly added to the crystallizer, with a 180 rev min^−1^ stirring rate for 1 h. After the OXA solids in the crystallizer dissolved completely, the mixed PDA-EtOH solution was slowly pumped at into the jacket using a peristaltic pump at 1 ml min^−1^ (model BT100-1L, Baoding Longer, China). With the addition of an alkaline solution, the precipitate was observed after about 25 min. The system was stirred for 2 h, then the anhydrous crystals were obtained after filtering and drying.

#### 2.2.2. Dihydrate

The PDA-OXA dihydrate was prepared by antisolvent crystallization in aqueous solution; EtOH was chosen as the anti-solvent (*v*_water_:*v*_EtOH_ = 1:5). Firstly, a total of 3.6 ± 0.1 g of PDA was dissolved in 10 g of water and then the solution was poured into a 100 ml double-jacketed crystallizer and agitated at a constant rate of 180 rev min^−1^ at ambient temperature. Then, 3.17 g of OXA powder was added to the PDA aqueous solution and continuously stirred until the mixed solution became transparent. Crystals of dihydrate gradually appeared with the addition of EtOH after about 15 min.

We also attempted to prepare the trihydrate products through a series of different proportions of highly saturated concentration PDA-OXA aqueous solutions by cooling crystallization; however, only the dihydrate was obtained. Finally, the structures of two crystalline products obtained were identified by powder X-ray diffraction (PXRD) for consistency.

### 2.3. Stability experiments

The stabilities of the anhydrate and dihydrate under different thermal and humidity conditions were studied. Relative humidity (RH) stability can also be called hygroscopicity, and is one factor to be assessed. 500 mg of solid powders of the two forms were placed in Petri dishes of varying RH (∼0, 32, 43, 67, 76, 98%) at room temperature (Yang *et al.*, 2016 ▸). After 1 week the powders were removed to record their quality change. After eight weeks, the samples were removed to carry out PXRD. The temperature was another factor to be investigated. In a similar manner, about 200 mg of crystalline products of the anhydrate and dihydrate, respectively, were weighed into several dishes, stored at room temperature, 40, 60, 80 and 90°C for one week, as well as at 110°C for 15 h. The samples were identified by PXRD after the test period.

### 2.4. Slurry experiments

Eleven solvents (methanol, ethanol, 1-propanol, 2-propanol, 1-butanol, ethyl acetate, acetone, aceto­nitrile, chloro­form, DMF and DMSO) were used for the phase transformations; the results can be used to evaluate the stability of the crystal in different solvent environments and then further determine the most stable form of PDA-OXA. 8 ml of each of the above organic solvents was poured into 10 ml vials and stirred using a rotor at 500 rev min^−1^. Then, an excess of the mixture of anhydrate and dihydrate of PDA-OXA (*m*:*m* = 1:1) was added into the different vials and then agitated for 1 week to reach a solid–liquid two-phase equilibrium. The turbid solution was filtered after the experiments and the solid obtained was dried and then characterized by PXRD. In particular, when slurried in the water–ethanol binary solvents with various water activities, the critical water activity required for the hydration of the two crystalline forms was determined at specified temperatures (Ding *et al.*, 2020 ▸). The water activity was calculated by the Margules formula (Li *et al.*, 2008 ▸), and temperatures of 10, 15 and 25°C were investigated. After filtering the mixed solution, the wet cakes were removed and analyzed by PXRD as well.

### 2.5. Single-crystal X-ray diffraction

Both the single crystals of the dihydrate and the trihydrate were obtained in pure water by cooling crystallization. A slight excess of crystalline powder of the dihydrate was added to 10 ml water, stirred thoroughly and completely dissolved at 50°C. The solution was cooled down slowly at a rate of 0.5°C h^−1^ to room temperature, then the quadrilateral dihydrate single crystal precipitated out after 1 h. Single crystals of trihydrate were obtained inadvertently while growing the dihydrate single crystals, which we have been unable to reproduce.

Crystallographic data of the two crystalline samples were collected at room temperature on a Bruker SMART APEX diffractometer with graphite-monochromated Mo *K*α radiation (λ = 0.71073 Å). The Bruker SMART-1000 program was used for data collection. Data collection and reduction were accomplished using the *SAINT* (Bruker, 2012 ▸) program through the *APEX3* software. The structures were analyzed by direct methods and refined by full matrix least-squares methods on *F* ^2^ (Sheldrick, 2015 ▸). The structure was solved and refined using *SHELXL-2018/3* (Usón & Sheldrick, 2018 ▸) through the graphical user interface *XSeed* (Atwood & Barbour, 2003 ▸). Measurement details of refinements processed using *PLATON* (Spek, 2003 ▸) are shown in Table 1 ▸. *Mercury* (version 3.3; Gavezzotti & Filippini, 1994 ▸) and *Olex2* (version 1.3; Dolomanov *et al.*, 2009 ▸) were used for structure visualization and the acquisition of standard diffraction patterns.

### 2.6. Powder X-ray diffraction

The crystallographic properties of samples were characterized by the Rigaku SmartLab diffractometer system using Cu *K*α radiation (λ = 1.5406 Å). Samples were scanned over the range 5° ≤ 2θ ≤ 40° in continuous scan mode, with a rate of 10° min^−1^ and a step size of 0.02°. The X-ray tube voltage and amperage were 45 kV and 40 mA, respectively. Furthermore, we tried to refine the PXRD patterns of the anhydrates with Pawley refinement and obtain the unit-cell parameters of the anhydrate single crystal; the relative parameters of the anhydrate are presented in Table S4 (Pekar *et al.*, 2021 ▸).

### 2.7. Spectroscopic analysis

Infrared spectra of the crystals were measured on a Nicolet iS5 FT-IR spectrometer with an ATR reflectance attachment for solid powders. Samples were analyzed over the range 4000−500 cm^−1^ with a resolution of 4 cm^−1^. A ReactRaman 785 spectrometer (METTLER TOLEDO) with a 785 nm optical fiber laser was used to capture the Raman spectra of crystals. The Raman spectra were collected in the range 200–2500 cm^−1^ with a resolution of 0.5 cm.

### 2.8. Thermal analysis

Thermogravimetric analysis (TGA) and differential scanning calorimetry (DSC) data were collected using a METTLER TGA/DSC 1 instrument with an alumina crucible under nitro­gen gas (flow rate 40 ml min^−1^) to characterize the thermal behaviors of the PDA-OXA crystalline samples. Approximately 3–5 mg of each sample was heated from 40 to 400°C at a constant heating rate of 10°C min^−1^. The crystals of anhydrate and dihydrate were characterized by polarized light hot-stage microscopy (HSM) on an Mshot MP41 polarizing microscope equipped with a digital camera (Mshot MS60) and a heating stage (Mshot K-3000B). The heating temperature ranges from room temperature to 220°C at a 2°C min^−1^ constant rate. All image captures were recorded for further analysis with 10 × 4 magnification.

### 2.9. Hirshfeld surface analysis

Molecular HSA and the associated fingerprint plots presented in this work were generated using the *CrystalExplorer* 17.5 software (Hirshfeld, 1977 ▸; Spackman & Jayatilaka, 2009 ▸; Spackman, 2013 ▸) to explore the intermolecular interactions in these molecular crystals, which are integral to understanding the overall packing modes (Turner *et al.*, 2017 ▸; Spackman & McKinnon, 2002 ▸). The normalized contact distance (*d*_norm_) surface was used for identification and quantification of intermolecular interactions that are relevant in the crystal lattice. Graphical plots of the Hirshfeld surfaces mapped over the *d*_norm_ function show a red–white–blue color scheme, where red and blue highlight shorter and longer contacts, respectively, while the white area indicates contacts at the van der Waals radii.

### 2.10. Computational details

The lattice energy calculation was performed using the *DMol* ^3^ module in *Materials Studio* (version 7.0; Accelrys, USA). Prior to calculations, the crystal structures were geometry-optimized. During the optimization, the unit-cell parameters were fixed. All calculations were performed using the generalized gradient approximation (GGA) by Perdew−Burke−Ernzerhof (PBE) (Singh & Banerjee, 2013 ▸) as exchange−correlation density functional and ultrasoft pseudopotentials with the addition of the Grimme D2 dispersion correction. DNP was chosen as the basis set before the calculations.

### 2.11. Polymerization experiments

#### 2.11.1. Melting polymerization

Bio-based nylon PA52 was synthesized as follows: the prepared PDA-OXA dihydrate salt was dissolved in deionized water (*m*_salt_:*m*_water_ = 1.5:1), then the transparent solution was poured into the autoclave (AC100ml, Beijing Century Senlong) and stirred. The temperature was raised to 180°C and maintained for 1 h. When the reaction was completed, the pressure in the autoclave was slowly reduced to atmospheric pressure within 1 h. Finally, the synthesized polymer was removed from the autoclave.

#### 2.11.2. Direct solid-state polymerization

In view of the fact that there is no solvent involved in direct solid-state polymerization (DSSP), the influence of the crystal forms of monomers on the polymerized product properties can be investigated. 10 g of PDA-OXA anhydrate and dihydrate monomer were weighed and added to the autoclave. Next, high-purity nitro­gen gas in the autoclave was replaced at least three times. The procedure of DSSP can be divided into four procedures: (1) the autoclave was heated to 150°C and the pressure inside the autoclave was kept at 0.5 MPa for 1 h. (2) The temperature was raised to 180°C and the pressure was kept at 0.8 MPa for 1 h. (3) The pressure inside the autoclave was reduced slowly to atmospheric pressure within 1 h and then maintained for 2 h, this step helps to increase the molecular weight of nylons. (4) Finally, the autoclave was opened and the final product was removed. The product was placed in a vacuum dessicator until the nylon product had completely dried, then characterization analysis could be carried out.

## 3. Results and discussion

### 3.1. Powder X-ray diffraction analysis

**Figure 2:**
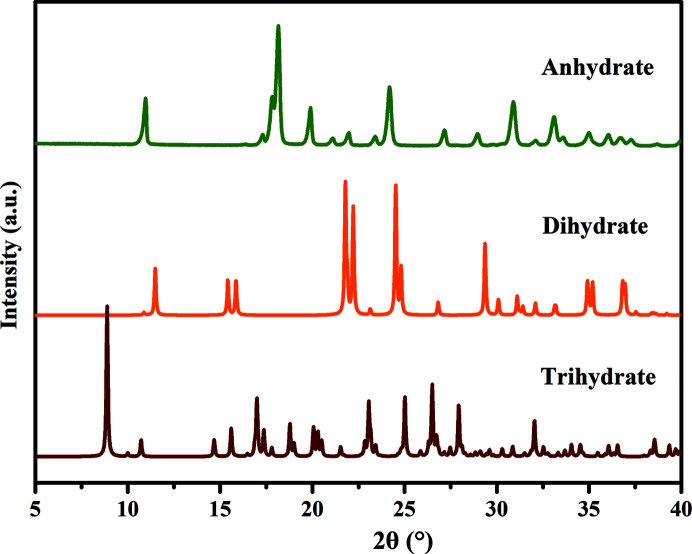
PXRD profiles for the three crystal forms of PDA-OXA.

**Figure 3:**
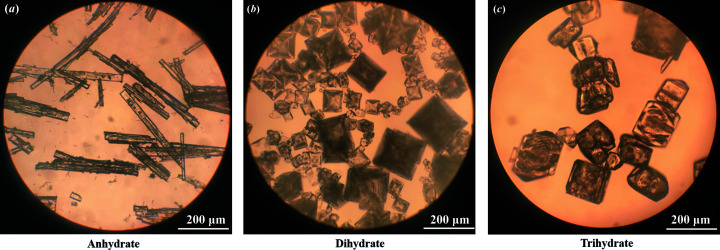
Optical micrographs of crystals obtained in experiments: (*a*) anhydrate, (*b*) dihydrate, (*c*) trihydrate. The magnifications are 16 × 4.

PXRD measurements were carried out to identify the crystalline phases and confirm the purity of the samples. The PXRD patterns of three PDA-OXA crystal forms are shown in Fig. 2 ▸. The fingerprint regions can be easily distinguished owing to their respective characteristic peaks. In addition, the experimental patterns confirm that the dihydrate sample is phase pure, similar to its single crystals (displayed in Fig. S1 of the supporting information). Notably, the three crystalline phases were also easily distinguished by their morphology: thin-rod-like for the anhydrate, cubic for the dihydrate and irregular blocks for the trihydrate, illustrated in Fig. 3 ▸. Note the photograph of the trihydrate was taken when the single crystal was obtained serendipitously.

### 3.2. FTIR and Raman analysis

FT-IR and Raman spectroscopy were both convenient tools for phase identification, used to gain further insight into the local structure of the two crystalline samples. Fig. S2 presents the IR spectrums of the anhydrate and dihydrate with markedly different spectral features. There is a discernible difference in the range 3000–3500 cm^−1^, which is the presence of the characteristic peak attributed to the OH stretching vibration at 3317.61 cm^−1^ of the dihydrate, whereas the anhydrate has no such peak (Jamieson *et al.*, 2006 ▸). The peak at 1615.95 cm^−1^ of the dihydrate and at 1596.27 cm^−1^ of the anhydrate both indicate the C=C stretching vibration of the benzene ring. Moreover, many other absorbing peaks in the fingerprint region (1300–400 cm^−1^) can be attributed to the hydrogen-bonding interactions between the N—H of PDA and the C=O of OXA. Thus, FTIR analysis can easily distinguish between the two crystalline phases of PDA-OXA. In addition, the Raman spectrum in Fig. S3 also shows notable identification ability. Three peaks of dihydrate under the pink area at 709.59, 1201.23 and 1720.08 cm^−1^ can be defined as the characteristic peaks, enabling rapid differentiation from the anhydrate.

### 3.3. Crystal structure analysis

A fundamental understanding of the molecular interaction types of the hydrates between the host crystal and water is essential to discern their effect on physicochemical properties such as solubility, dissolution rate and stability (Li *et al.*, 2021 ▸). In order to clarify the effect of water on the crystal structures and the reason the dihydrate is more stable than the trihydrate, a summary of the crystallographic details of the dihydrate and trihydrate is given in Table 1 ▸, and the selected bond lengths and angles are summarized in Table S1 of the supporting information; the data show significant differences in terms of crystal system, unit volume, crystal density *etc.* Moreover, the refinement parameters and the fitted diffraction pattern of the anhydrate are presented in Table S2 and Fig. S4, respectively.

#### 3.3.1. Dihydrate

**Figure 4:**
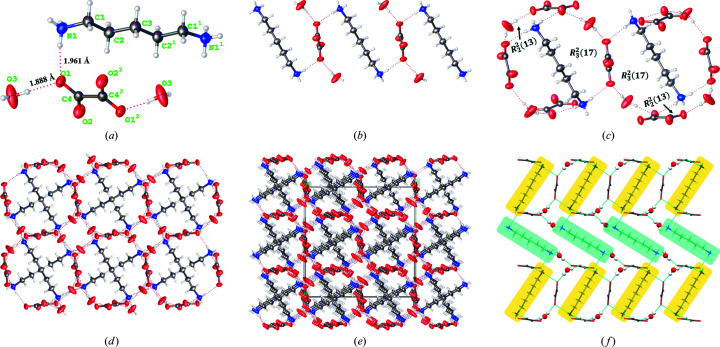
(*a*) Asymmetric unit with the selected atom-labeling scheme of the dihydrate. The ellipsoids are shown at 50% probability. (*b*) Crystal structure of a dinuclear unit of the dihydrate. (*c*) View of the packing structure of the dihydrate with hydrogen bonds between the coordinated water and PDA-OXA (dashed lines). (*d*) 3D supramolecular framework with open 1D channels and and (*e*) packing diagrams along the *b* axis, respectively. (*f*) The consistency of the ‘voids’ and the reverse arrangement of PDA molecules shown in different colors.

The dihydrate is a colorless cubic crystal. The asymmetric unit consists of one molecule each of PDA and OXA and two water molecules, which crystallize in the space group *I*4_1_/*a*. The asymmetric unit, dinuclear unit, packing modes and 3D supramolecular frameworks of the dihydrate are shown in Fig. 4 ▸.

The close N—H⋯O and O—H⋯O contacts [1.961 and 1.888 Å, Fig. 4 ▸(*a*)] indicate strong hydrogen-bonding interactions between the PDA, OXA and water molecules. Note that the smallest asymmetric unit is only half of the asymmetric unit (depicted in Fig. S5), which can be attributed to the fact that the unit cell of the dihydrate is centrosymmetric. In the dihydrate double layers [Fig. 4 ▸(*b*)], each OXA anion interacts with two PDA cations and two symmetrically equivalent water molecules owing to the hydrogen bonding between the carb­oxy­lic and amino functional groups. In Fig. 4 ▸(*c*), based on the double layers, the water molecules act as a bridge to connect two adjacent OXA anions through individual carboxyl groups, establishing two 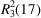 and 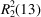 supramolecular graph sets. Figs. 4 ▸(*d*) and 4 ▸(*e*) are the 3D supramolecular and packing frameworks of the dihydrate along the *b* axis, respectively. Notably, there are numerous dinuclear units that alternate in opposite directions, forming a spectacular tight football-shaped network; this close ‘graphene-like’ structure might provide the dihydrate with enhanced stability. Moreover, the square ‘voids’ in the middle of four ‘footballs’ are the result of hydrogen-bonding interactions between the dinuclear unit and its anti-adjacent PDA and water molecules, illustrated in Fig. 4 ▸(*f*). The PDA molecules in the yellow area are connected to the OXA molecules whereas those in the green area are connected to the water molecules.

#### 3.3.2. Trihydrate

**Figure 5:**
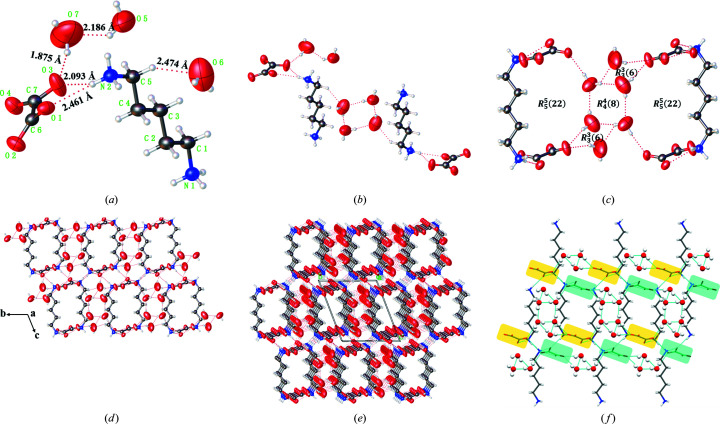
(*a*) Asymmetric unit with the selected atom-labeling scheme for the trihydrate. The ellipsoids are shown at 50% probability. (*b*) Crystal structure of a dinuclear unit of the trihydrate. (*c*) View of the packing structure of the trihydrate with hydrogen bonds between the coordinated water and PDA-OXA (dashed lines). (*d*) and (*e*) 3D supramolecular framework of trihydrate with an open 1D channel and packing diagrams along the *a* axis, respectively. (*f*) The reverse arrangement of OXA molecules shown in green and yellow.

The trihydrate crystallizes as a block crystal which is similar in shape to the dihydrate; its single-crystal structure was successfully parsed. The crystals belong to a triclinic system with the space group *P* 1; Fig. 5 ▸ illustrates the intermolecular interactions, hydrogen-bonded synthons and packing networks of the PDA-OXA trihydrate. In Fig. 5 ▸(*a*), there are three water molecules around the host PDA and OXA ions in the asymmetric unit. Among them, one is bound by the O7—H⋯O3 interaction (1.875 Å) with the carb­oxy­lic group of OXA, another by the C5—H⋯O6 contacts (2.474 Å) and the third via the first water molecule as its hydrogen bond acceptor, connecting through the O5—H⋯O7 interaction (2.186 Å). In particular, we can see that two host molecules form an intramolecular interaction according to the interactions between the N2 atom on the amino group of PDA with the two O atoms on the carboxyl group of OXA (N2—H⋯O3, 2.093 Å; N2—H⋯O1, 2.461 Å). Fig. 5 ▸(*b*) is the crystal structure of the dinuclear unit of the trihydrate and Fig. 5 ▸(*c*) shows the packing view of the two dinuclear units. The two host molecules connected with two water molecules form an 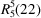 synthon. Also, two 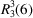 and one 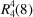 hydrogen bonding synthons comprise the six water molecules of two contiguous asymmetric units, bridging two neighboring dinuclear units. Figs. 5 ▸(*d*) and 5 ▸(*e*) show the packing networks of the trihydrate along the *a* axis based on the abundant intermolecular synthons. Additionally, Fig. 5 ▸(*f*) depicts the OXA molecules stacked in an alternate anti-parallel arrangement, marked in green and yellow.

Table 2 ▸ shows the torsion angle comparison of the two PDA-OXA hydrates, which can be attributed to the different intermolecular interactions and stacking modes.

#### 3.3.3. Anhydrate

Although we failed to obtain obtain single crystals of the anhydrate failure, the related crystalline information collected through the Pawley refinement from its PXRD pattern was also investigated and listed in Table S2. The PDA-OXA anhydrate is a thin rod-like crystal with a monoclinic system and the space group *C*2/*m*. Both refinement parameters were <10 (*R*_wp_ = 9.03%, *R*_p_ = 6.61%), indicating the refinement results are accurate and reliable (Fig. S4).

Although water molecules play a key role in the crystal packing of the dihydrate and trihydrate (Fig. S6), which are tightly packed in the lattice by hydrogen bonding, both hydrates are not channel-hydrates, as confirmed from observation and analysis of the Connery surfaces highlighted in Fig. S7. Furthermore, the hydration layer composed of water molecules will also have a great influence on the stability of the crystal. According to the density rule (Bernstein, 2007 ▸), the crystal form with the higher density involves better crystal packing and higher stability. Fig. S8 shows the comparison of crystal density (1.329 g cm^−3^ for the dihydrate and 1.265 g cm^−3^ for the trihydrate) and packing coefficient (0.7038 for the dihydrate and 0.6609 for the trihydrate) between the two hydrates, it seems the dihydrate may be more stable from the perspective of these two factors. To more intuitively describe the intermolecular interactions of the two crystal forms, further analysis of the Hirshfeld surface was carried out (Spackman & Jayatilaka, 2009 ▸).

### 3.4. Hirshfeld surface analysis

HSA was carried out to aid a more quantitative understanding of the molecular packing and contributions of the main intermolecular interactions that are responsible for the crystal stabilization. Fingerprint plots further quantified the level of contact interactions between molecules by highlighting particular close contacts (Zhang *et al.*, 2017 ▸). The brighter red spots that appear on the *d*_norm_ surface usually correspond to the stronger closer contacts of N—H⋯O and O—H⋯O hydrogen bonds, whereas the less bright spots represent some weaker hydrogen bonds, such as van der Waals interactions.

**Figure 6:**
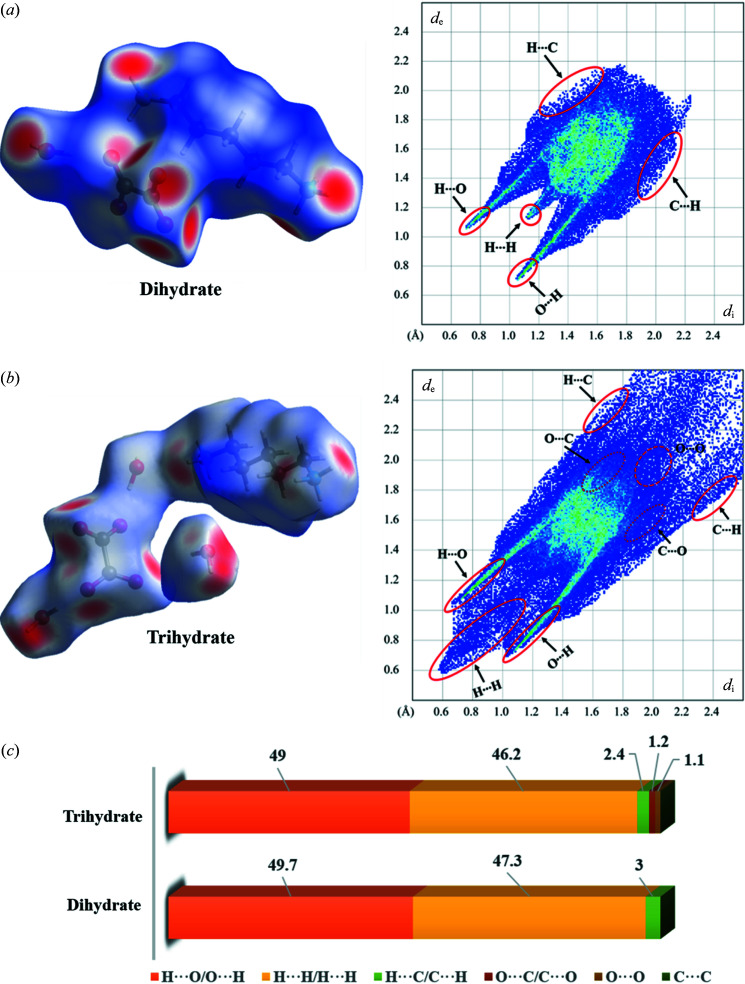
Hirshfeld surface mapped over *d*_norm_ in the color range −0.6615 to 1.1043 a.u./−0.8808 to 2.2997 a.u. showing the intermolecular close contacts in the (*a*) dihydrate and (*b*) trihydrate. (*c*) Relative percentage contributions to the calculated Hirshfeld surfaces for the various close intermolecular contacts in the PDA-OXA dihydrate and trihydrate.

As shown in Fig. 6 ▸, the HSA maps of the two PDA-OXA hydrates indicate graphically the closer hydrogen-bonding contacts and their corresponding fingerprints. Notably, O⋯H/H⋯O contacts are visible as a pair of symmetrical ‘wing-like’ sharp spikes whereas the H⋯H contacts look like the ‘head’ in the middle of the wings. The remaining relatively weak interactions appear on the sides of the surface edges. For further clarity, more detailed information about the HSAs of the dihydrate and trihydrate are shown in Figs. S9 and S10, as well as the specific hydrogen bonds and van der Waals interactions plotted in Fig. S11.

Fig. 6 ▸(*c*) depicts the percentage contribution of the intermolecular close contacts. O⋯H/H⋯O contacts in the hydrates, which contribute the largest percentage (49.7% versus 49.0%) of the total interactions. It is likely that the H⋯H contacts of the hydrogen-bond skeleton also account for the majority of the interactions (47.3% for the dihydrate and 46.2% for the trihydrate). In addition, the C⋯H interactions are another important contact but vary with the multicomponent-specific crystal structure. Yet compared with the dihydrate, the consistency of the trihydrate is more complicated owing to some weak interactions such as C⋯O/O⋯C and O⋯O contacts. Nevertheless, concerning the main contribution of hydrogen-bonding interactions, we can speculate that the dihydrate may have a higher stability than the trihydrate owing to the support of the stronger hydrogen bonds, this phenomenon is consistent with the results of the above comparison of crystal density.

### 3.5. Computational analysis

To further evaluate the above assumption, we calculated the lattice energies of the two hydrates, which are depicted schematically in Table S3. As expected, the dihydrate shows the highest lattice energy (−280.98 kcal mol^−1^), whereas that of the trihydrate is lower (45.03 kcal mol^−1^) than the former (difference −235.95 kcal mol^−1^). Moreover, the binding energy of the dihydrate is superior to that of the trihydrate (−26498.19 kcal mol^−1^ versus −7015.44 kcal mol^−1^), the former being about 3.8 times greater than the latter. This large energy difference demonstrates that there is a great nucleation barrier for the trihydrate, which makes it impossible to obtain the crystalline product of the trihydrate through conventional crystallization experiments. The calculation results confirmed our inference and provide a convincing explanation of why the dihydrate is more stable than the trihydrate and why the trihydrate is very difficult to obtain.

From the results of the HSA and energy calculations, we concluded that heteromolecular N—H⋯O and O—H⋯O hydrogen bonds between PDA and OXA play a key role in the construction of the crystal structures, especially the dihydrate.

### 3.6. Thermal analysis

**Figure 7:**
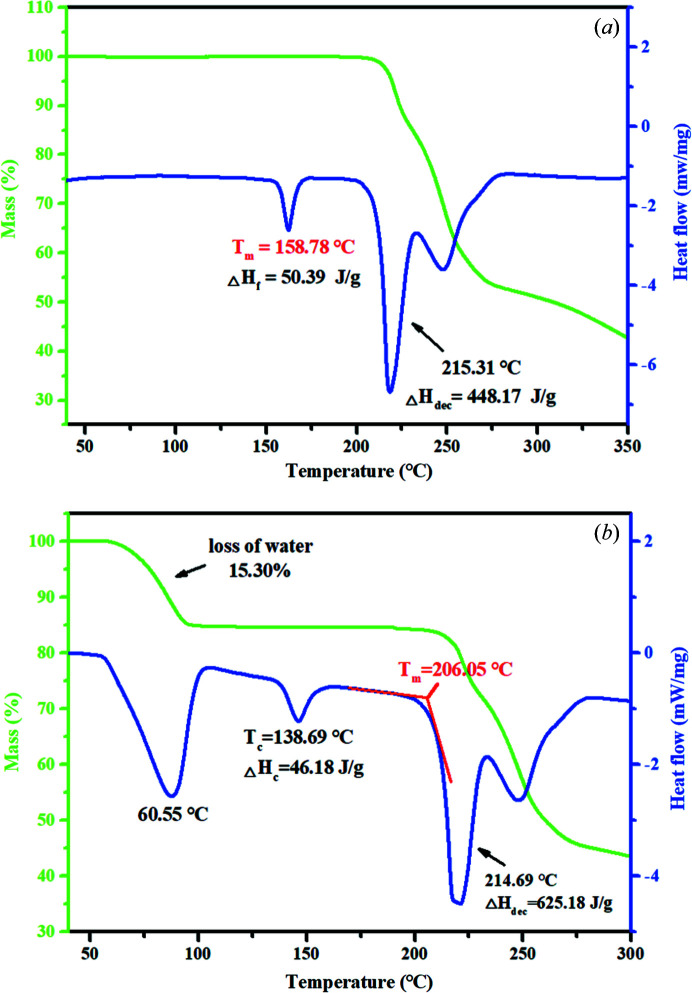
TGA and DSC curves of the (*a*) anhydrate and (*b*) dihydrate of PDA-OXA.

The thermal stability of the anhydrate and dihydrate crystalline powders were investigated by TGA and DSC measurements. As shown in Fig. 7 ▸(*a*), the anhydrate of PDA-OXA shows no weight loss before its melting point (158.78°C) whereas the dihydrate shows a total mass loss of 15.3%, starting at approximately 60.55°C in the DSC curve in Fig. 7 ▸(*b*), which corresponds to two water losses (theoretical: 15.77%). Note, there is a short peak (138.69°C) on the dihydrate DSC curve, which is not the melting temperature, but is in fact a transcrystallization temperature arising from the phase transformation from the dihydrate to a new metastable anhydrate. This phenomenon has been confirmed by PXRD measurements for the sample after thermal dehydration (see Fig. S12). An obvious endothermic peak occurs around 215°C for both crystalline forms, along with a major weight loss, which may be attributed to a combined effect of melting and decomposition. The near-identical decomposition temperature also clarifies that the crystal structures of the two samples are consistent at this point, indicating that the crystal structure of the anhydrate collapsed and began to reorganize after the melting temperature, and then transformed into the same metastable crystal as the dihydrate.

**Figure 8:**
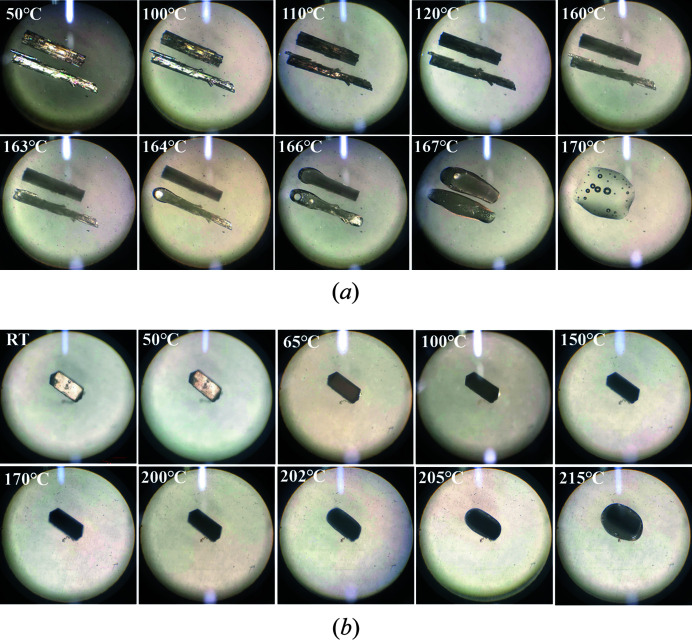
HSM pictures of the (*a*) anhydrate and (*b*) dihydrate crystals of PDA-OXA.

To gain insight into the molecular organization of the crystal structures, the melting process of the anhydrate and dihydrate (illustrated in Fig. 8 ▸) were analyzed by polar light hot-stage microscopy in detail.

The results observed from melting were in accordance with the TGA-DSC thermal analysis. As seen in Fig. 8 ▸(*a*), the anhydrous powders became opaque when the temperature exceed 110°C. As the temperature increased, the crystal started to melt at 160°C, which is approximately equal to its melting point. The phenomenon in Fig. 8 ▸(*b*) explains the transcrystallization behavior in the thermal analysis of the dihydrate, as no melting signs occurred around 150°C. Herein, the peak at 138.69°C is not related to the melting but is related to the phase transformation. What is more, we can see that the dihydrate crystal began to melt at 205°C, which supports the results of the thermal analysis (206.05°C).

### 3.7. Stability analysis

Hygroscopicity experiments whereby the PDA-OXA dihydrate and anhydrate were subjected to various humidities in the surroundings were performed, and the results are given in Fig. S13. Both crystalline solid powders were stable when RH ≤ 76%. But when the humidity was increased up to 98%, in all the forms emerged an obvious moisture absorption, manifested in a sharp quality change (188.87% for the anhydrate versus 146.78% for the dihydrate). The dihydrate showed a lower mass change compared with the former, indicating that the dihydrate has better stability under high humidity. Besides, uncommon steps appear on the moisture absorption curve of the anhydrate (RH = 98%) which can be explained by the macroscopic changes on the crystal surface during the hygroscopic process. As recorded in Fig. S14, almost all anhydrous solid powders absorbed moisture and then dissolved after 2 weeks [Fig. S14(*c*)], accompanied by the formation of block dihydrate crystals. Therefore, this stage was caused by the growth of the dihydrate single crystals. In this situation, the dihydrate crystals continued to absorb moisture until they completely dissolved.

**Figure 9:**
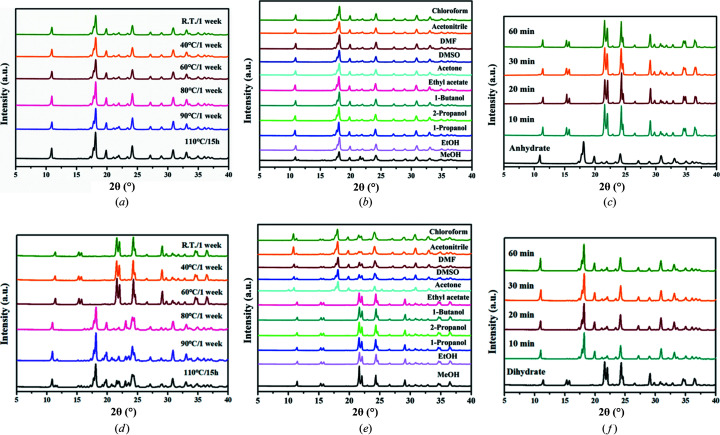
SST and slurry results for the (*a*)–(*c*) anhydrate and (*d*)–(*f*) dihydrate of PDA-OXA under different temperature and solvent conditions.

The effects of temperature on the stability and the transition between the two PDA-OXA phases were demonstrated by the SST and slurry experiments. Figs. 9 ▸(*a*) and 9 ▸(*d*) show the stability at different temperatures for the anhydrate and dihydrate, respectively. We can see that the anhydrate exhibits high thermal stability and maintains its structure even at 110°C for 15 h. Similarly, the anhydrate remains stable in different solvents through the slurry transformation [Fig. 9 ▸(*b*)]. In contrast, the dihydrate converted to the anhydrate when the temperature was raised to 80°C for 1 week [Fig. 9 ▸(*d*)]. Fig. 9 ▸(*e*) shows that the dihydrate undergoes desolvation in chloro­form, aceto­nitrile, DMF, DMSO and acetone whereas it remains stable in the rest of the solvents. Furthermore, the transformation results of Figs. 9 ▸(*c*) and 9 ▸(*f*) indicate that both the two crystalline forms can achieve mutual conversion easily. If the anhydrous solid powder is placed in pure water for 10 min, then it will transform to the dihydrate; when the dihydrate crystals are heated to 110°C for 10 min, the anhydrate can be obtained.

According to the above observed phenomenon, it is clear that the transformation between the anhydrate and dihydrate of PDA-OXA is reversible under the appropriate conditions. In particular, based on the crystal structure, hygroscopicity analysis and thermal stability, we can conclude that the dihydrate of PDA-OXA is more stable than the other two crystal forms.

### 3.8. Transitions between the dihydrate and anhydrate

We propose that single crystals of the anhydrate are very hard to obtain at the onset, shown as the flocculent crystals obtained by slow evaporation in pure solvents; the crystals are all poor in quality and cannot meet the detection standard. At the same time, the method of binary solvents is also not feasible. Once water is involved, all forms obtained are the dihydrate. To further shed light on the phenomenon, the relative stability and transformation of the two PDA-OXA crystal forms under varying temperature and water activity, two experiments under various thermodynamic conditions were designed and performed, the slurry experiments are in a water–ethanol binary system.

**Figure 10:**
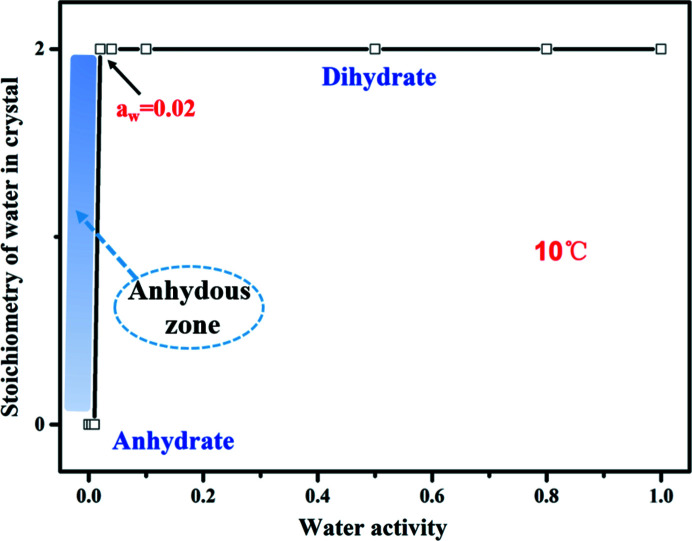
Phase diagram of the water activity of the PDA-OXA dihydrate and anhydrate at 10°C, which determined the boundary of transformation between the dihydrate and anhydrate zones.

The phase diagram after total equilibration for 24 h was measured to evaluate the dependence of PDA-OXA on water activity (*a*_w_) in ethanol–water mixtures at different temperatures, the results are depicted schematically in Figs. 10 ▸ and S15. As shown, even if the situation has been controlled from 25°C *a*_w_ = 0.07, down to 10°C *a*_w_ = 0.02, the anhydrate is still converted to the dihydrate. The area of the anhydrate zone is extremely narrow (shown in Fig. 10 ▸, marked by the blue strip) and the hydrate zone is rather widespread in this case. According to the above results, we think that the narrow area for the anhydrate is the fundamental reason why its single crystal is so difficult to obtain.

Above all, the mutual transformation relationship of the PDA-OXA anhydrate and dihydrate can be concluded in Scheme 1, taking into account all of the above transformation experiments.[Chem scheme1] 
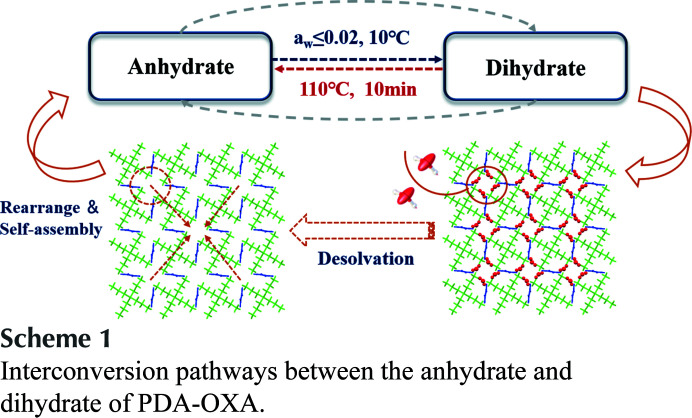


### 3.9. Polymerization analysis

**Figure 11:**
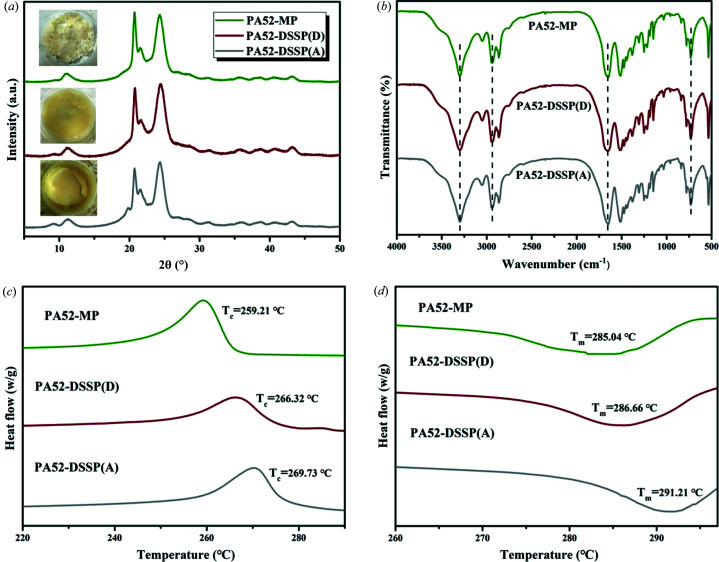
(*a*) PXRD and (*b*) FTIR characterization analyses of the PA52 products. Thermodynamic analysis of the nylons, comparing the (*c*) recrystallization temperature and (*d*) melting temperature by the MP and DSSP methods.

Polymerization experiments were carried out using the prepared anhydrate and dihydrate crystalline products as nylon raw materials. The traditional melting polymerization (MP) and DSSP were used to compare the thermodynamic properties of the nylon products (PA52) obtained by different polymerization methods. Furthermore, the effects of raw materials with different crystalline forms on the properties of the polymerized products were also illustrated under the conditions of the DSSP method. Through qualitative analysis, thermodynamic analysis and molecular weight determination (Gel Permeation Chromatography, GPC, Rid-20A), the performance of the nylons obtained were further determined and compared. The results are displayed in Figs. 11 ▸ and S16. Note that the mark of DSSP(D) and DSSP(A) refer to the use of dihydrate and anhydrate as monomers in DSSP, respectively.

Fig. 11 ▸(*a*) shows the PXRD patterns of the PA52 nylon products by the MP and DSSP methods, the peaks on the patterns are almost identical, as well as for the FTIR analysis [Fig. 11 ▸(*b*)], indicating that the nylon products obtained by different polymerization methods are the same. Notably, graphs of the actual polymers are embedded in their corresponding PXRD patterns. The color of the product obtained by the two polymerization methods is light, indicating the effect of the two polymerizations is relatively ideal. In addition, TGA-DSC analysis of the three nylons was also performed as follows. Figs. 10 ▸(*c*) and 11 ▸(*d*) illustrate that fact that the thermodynamic properties of the PA52 products obtained are different, even though they have the same structure. It appears that the product prepared by DSSP with the anhydrous powders as the raw monomers show the highest thermal stability, owing to its melting temperature (*T*_m_) of 291.21°C (recrystallization temperature *T*_C_ = 269.73°C). The following is prepared by DSSP with the dihydrate crystalline solid as the raw monomer, with a melting temperature (286.66°C) that is a little lower than the former. By contrast, the MP method is less effective than DSSP for the MP nylon products and has the lowest melting temperature (285.04°C). Despite the melting points of the three PA52 products being slightly different, all of them exceed 280°C, thus, it can be said that nylon 52 is a new type of high-temperature resistant nylon.

For further clarity on the polymerization performance, the molecular weight determination of the products was calculated and is listed in Table 3 ▸. We can see that all the nylon products have a similar number average molecular weight (*M*_n_) and weight average molecular weight (*M*_w_). In particular, the *M*_w_ > 20 000 for PA52, indicating excellent polymerization effects. Based on the above observation, we can conclude that PA52 is a new type of bio-based high-temperature nylon with practical industrial application prospects.

## 4. Conclusions

To summarize, we found that PDA-OXA exists in three crystalline forms. One is the cubic dihydrate, another is the rod-like anhydrate and the last is the trihydrate. We investigated and characterized their crystal structures, intermolecular interactions, thermal stabilities and phase transformation behaviors by various analytical techniques such as and slurry experiments.

Moreover, a fully comprehensive analysis of the difference in the crystal conformation and hydrogen-bonding interactions between the dihydrate and the trihydrate was carried out using powerful HSA and lattice energy calculation tools. The results provide a good explanation for why the crystal products of the trihydrate are difficult to produce, which is mainly due to the trihydrate having less hydrogen bonding and a lower lattice energy compared with the dihydrate. The large energy barrier makes it extremely difficult to precipitate. Moreover, the relative stability relationship between the dihydrate and the anhydrate was further established. The stability difference between the two hydrates is well understood through the establishment of the structure–property relationship.

The hygroscopicity and the thermodynamic properties together prove that the stability of the dihydrate is higher than that of the anhydrate. The transformation mechanism between the two crystalline forms is easy to understand, which was mainly dominated by the temperature or the surrounding RH. The dihydrate can quickly transform to the anhydrate at 110°C after 10 min whereas the anhydrate can undergo transformation easily when the water activity is >0.02 (10°C). We think that the narrow survival area for the anhydrate is the fundamental reason why its single crystal is so difficult to obtain.

Finally, MP and DSSP are two polymerization methods for PA52 using either the anhydrate or the dihydrate as the monomers; all the nylon products show improved morphology, molecular weight and thermal stability. According to the thermodynamics analysis, the polymorphism of the monomer has a significant effect on the thermal properties of the nylon products; using the anhydrate as the monomer provides better thermodynamic properties than using the dihydrate. To our knowledge, the nylon 52 products obtained are examples of a new bio-based high-temperature nylon with prospective practical industrial applications.

## Supplementary Material

Crystal structure: contains datablock(s) trihydrate, dihydrate. DOI: 10.1107/S2052252522010442/lq5048sup1.cif

Structure factors: contains datablock(s) dihydrate. DOI: 10.1107/S2052252522010442/lq5048sup2.fcf

Structure factors: contains datablock(s) trihydrate. DOI: 10.1107/S2052252522010442/lq5048sup3.fcf

Supporting figures and tables. DOI: 10.1107/S2052252522010442/lq5048sup4.pdf

CCDC references: 1917787, 2044247

## Figures and Tables

**Table 1:** Detailed crystallographic data for the PDA-OXA dihydrate and trihydrate

	Dihydrate	Trihydrate
Empirical formula	C_7_H_16_N_2_O_4_·2H_2_O	C_7_H_16_N_2_O_4_·2.5H_2_O
Formula weight	228.25	237.36
Crystal system	Tetragonal	Triclinic
Space group	*I*4_1_/*a*	*P* 1
*a* (Å)	16.2220 (14)	6.849 (5)
*b* (Å)	16.2220 (14)	9.484 (7)
*c* (Å)	8.7239 (7)	10.764 (8)
α (°)	90	110.712 (8)
β (°)	90	97.505 (8)
γ (°)	90	91.358 (8)
Volume (Å^3^)	2295.7 (4)	646.6 (8)
*Z*	8	2
*D*_calc_ (g cm^−3^)	1.321	1.265
μ (mm^−1^)	0.114	0.112
*F*(000)	992	258
Crystal size (mm)	0.30 × 0.33 × 0.40	0.14 × 0.30 × 0.45
Observed data [*I* > 2σ(*I*)]	799	1635
*R*_int_	0.055	0.029
*R*_1_ [*I* > 2σ(*I*)]	0.047	0.073
*wR* ^2^	0.115	0.231
GOF on *F* ^2^	1.06	1.07
Difference density (e Å^−3^)	−0.19, 0.18	−0.49, 0.32
CCDC	2044247	1917787

**Table 2:** Comparison of the torsion angle data for the dihydrate and trihydrate

Dihydrate	Torsion angle (°)	Trihydrate	Torsion angle (°)
N1–C1–C2–C3	−174.88 (16)	N1–C1–C2–C3	−175.9 (2)
C1–C2–C3–C2†	−178.90 (17)	C1–C2–C3–C4	178.8 (2)
O1–C4–C4 –O1‡	−180.0 (2)	C2–C3–C4–C5	−179.9 (2)
O1–C4–C4–O2	−1.0 (3)	C3–C4–C5–N2	178.7 (2)
O2–C4–C4–O1†	1.0 (3)	O1–C6–C7–O3	23.5 (4)
O2–C4–C4–O2	−180.0 (2)	O2–C6–C7–O3	−156.2 (3)
		O1–C6–C7–O4	−156.6 (2)
		O2–C6–C7–O4	23.7 (3)

† Symmetry code: 1 − *x*, 1/2 − *y*, *z*.

‡ Symmetry code: 1 − *x*, 1 − *y*, 1 − *z*.

**Table 3:** Comparison of the molecular weight and thermodynamic properties for PA 52 by MP and DSSP methods

	*M*_n_ (Da)	*M*_w_ (Da)	Polydispersity	*T*_melt_ (°C)	*T*_recrystal_ (°C)
PA52-MP	10247	22527	2.20	285.04	259.21
PA52-DSSP(D)	10537	24888	2.36	286.66	266.32
PA52-DSSP(A)	11071	22392	2.02	291.21	269.73
