# Supplementary material for: The effect of polymorphism on polymer properties: crystal structure, stability and polymerization of the short-chain bio-based nylon 52 monomer 1,5-pentanedi­amine oxalate
Source: IUCrJ. 2023 Jan 1;10(Pt 1):52–65. doi: 10.1107/S2052252522010442 (PMC9812218; doi:10.1107/S2052252522010442)

IUCrJ

Volume 10 (2023)

Supporting information for article:

The effect of polymorphism on polymer properties: crystal structure, stability and polymerization of the short-chain bio-based nylon 52 monomer 1,5-pentanediamine oxalate

Zihan Li, Shushu Li, Pengpeng Yang, Xincao Fang, Qingshi Wen, Ming Li, Wei Zhuang, Jinglan Wu and Hanjie Ying

Figure S1 Comparison of PXRD patterns with the simulated patterns from single-crystal X-ray diffraction (SCXRD) of PDA-OXA dihydrate.

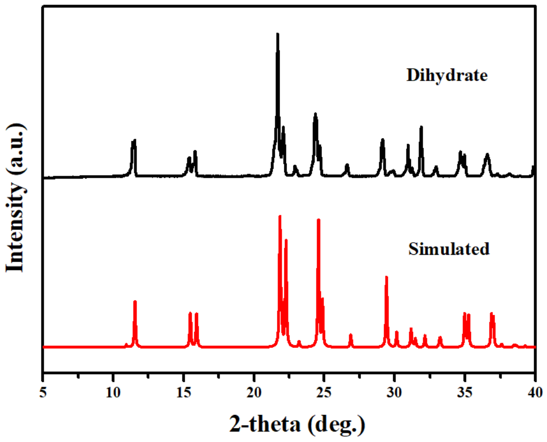

Table S1 Hydrogen bonds data of the PDA-OXA two crystal forms.

D—H···A	D(D-H) / Å	d(H···A) / Å	d(D···A)/ Å	D—H···A
**Dihydrate**				
N1—H1A···O1[a]	0.8900	1.9800	2.842(2)	163.00
N1—H1B···O3[b]	0.8900	1.8800	2.695 (2)	151.00
N1—H1C···O1[c]	0.8900	2.3700	3.026(2)	131.00
N1—H1C···O2[d]	0.8900	2.0000	2.827(2)	154.00
O3—H3E···O1	0.8500	1.9100	2.756(2)	178.00
O3—H3F···O2[e]	0.8500	1.9300	2.783(2)	178.00
Symmetry Code at atoms: a=x, y,1+z; b=1/2-x,1-y,1/2+z; c=3/4-y,1/4+x,5/4+z; d=-1/4+y,5/4-x,5/4-z. e=-				
**Trihydrate**				
D—H···A	D(D-H) / Å	d(H···A) / Å	d(D···A)/ Å	D—H···A
N1—H1A···O1[1]	0.8900	1.9300	2.814(3)	174.00
N1—H1B···O2[2]	0.8900	1.9500	2.829(4)	171.00
N1—H1C···O2[3]	0.8900	2.3800	3.043(4)	131.00
N1—H1C···O4[3]	0.8900	2.0400	2.861(4)	152.00
N2—H2A···O1[4]	0.8900	2.4600	3.197(4)	141.00
N2—H2A···O3[4]	0.8900	2.0900	2.869(4)	145.00
N2—H2B···O1[5]	0.8900	1.9700	2.855(4)	171.00
N2—H2C···O4[6]	0.8900	1.9200	2.808(4)	174.00

O5-H5E···O2	0.8500	2.0500	2.898(5)	171.00
O5-H5F···O6[7]	0.8500	1.8600	2.707(7)	171.00
O6-H6C···O3[8]	0.8500	2.0300	2.858(6)	165.00
O6-H6D···O5[9]	0.8500	2.0900	2.922(7)	166.00
O7-H7C···O3	0.8500	1.8700	2.706(14)	170.00
O7-H7D···O5[2]	0.8500	1.9900	2.826(16)	170.00
C5-H5A···O6[4]	0.9700	2.4800	3.332(6)	146.60

Symmetry Code at atoms: 1=1-x,1-y,1-z; 2=x,1+y, z; 3=2-x,1-y,1-z; 4=x, y, -1+z; 5=1-x,1-y, -z; 6=-1+x, y, -

7=1+x, -1+y, z; 8=-1+x, y, z; 9=1-x,1-y,2-z.

Figure S2 FTIR spectroscopy characterization of the anhydrate (black) and dihydrate (red).

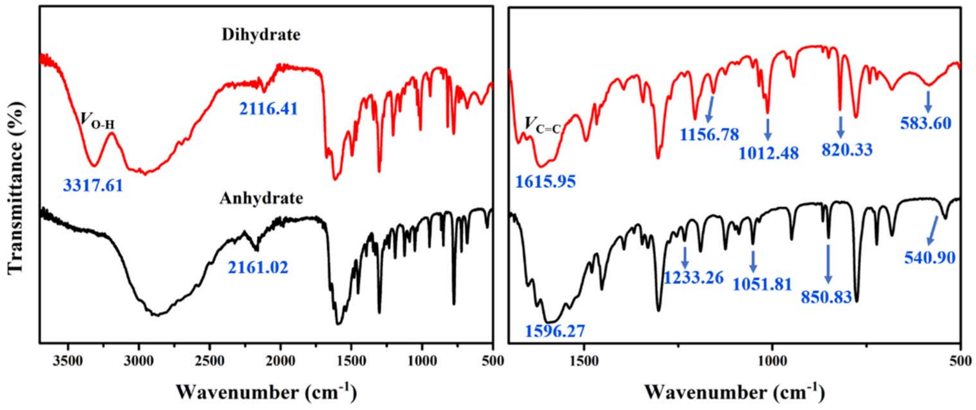

Figure S3 Raman spectroscopy of the anhydrate (orange) and dihydrate (green).

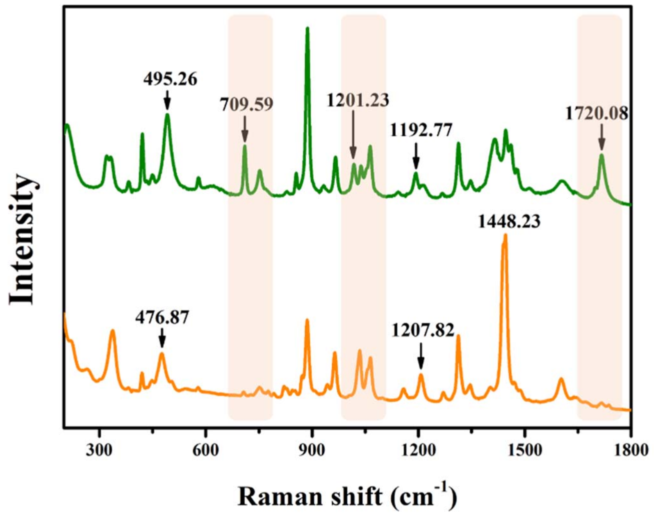

Table S2 Crystallographic parameters obtained from Pawley refinement for PDA-OXA anhydrous forms.

	Anhydrous form
Empirical formula	$C_7H_{16}N_2O_4$
Formula weight	192.21
Crystal system	Monoclinic
Space group	*C2/m*
a (Å)	10.2905
b (Å)	6.0101
c (Å)	9.8982
α (deg)	90
β (deg)	124.947(4)
γ (deg)	90

Figure S4 Diffraction pattern of anhydrate at ambient conditions. The observed pattern (circle), the best fit profile (line) and the difference curve between the observed and calculated profiles (green short lines) are shown.

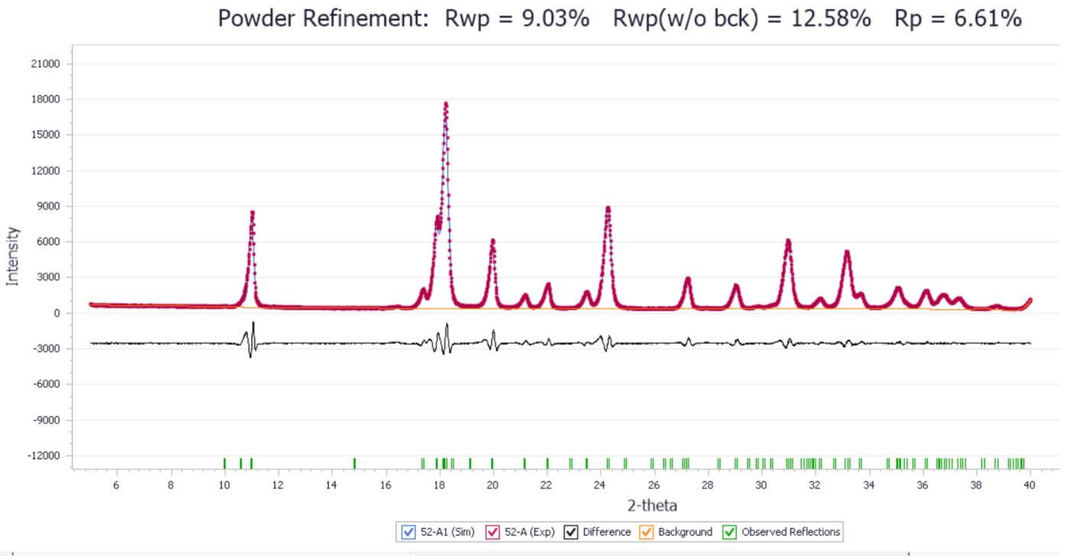

Figure S5 (a) Structure of the smallest asymmetric unit of dihydrate. (b) An inversion center is shown as a yellow point located between the two C4^{I} atoms of the OXA anion and a 2-fold inversion axis is shown as a green line through the C3^{II} atom of the PDA cation. Symbol: (I): 1-x, 1-y, 1-z; (II): 1-x, 1/2-y, z.

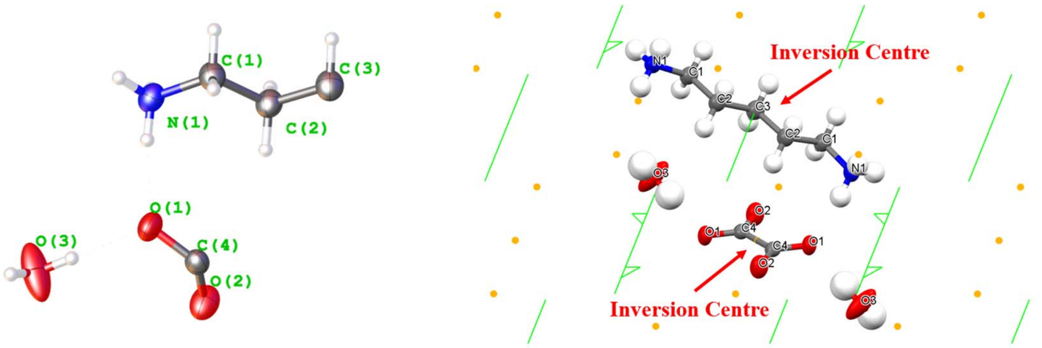

(a) (b)

Figure S6 Packing view of the water molecules in the structures of (a) dihydrate and (b) trihydrate. For a further clear analysis, the water molecules are set in spacefill mode.

(a) (b)

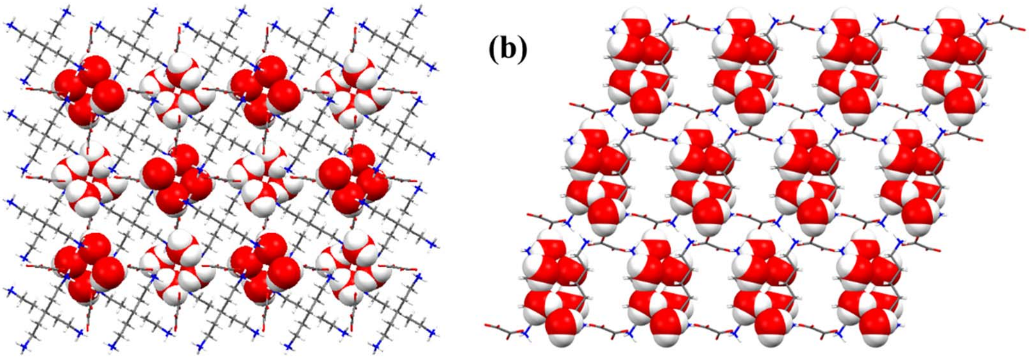

Figure S7 Connolly surfaces of the PDA-OXA dihydrate (a) and trihydrate (b).

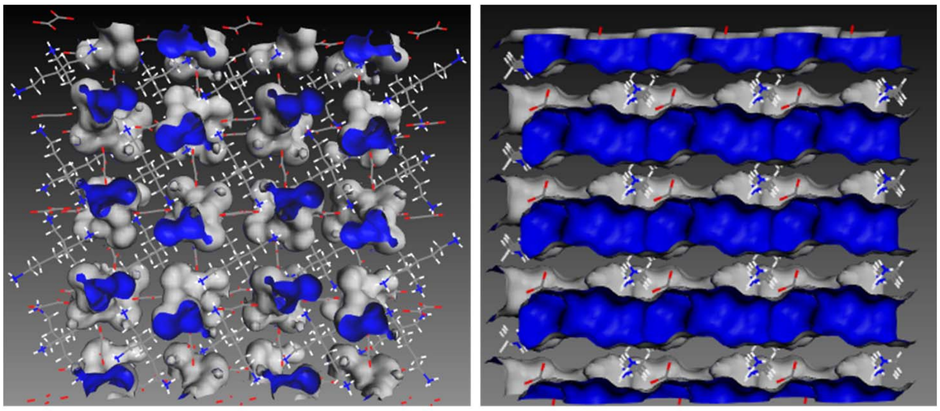

(a) (b)

Figure S8 Comparation of the PDA-OXA two hydrates in terms to packing coefficient and crystal density.

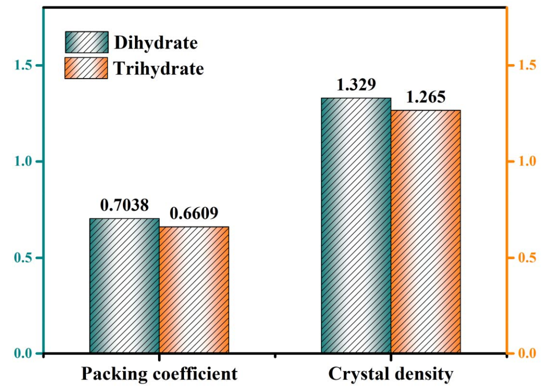

Figure S9 Hirshfeld surface and its corresponding fingerprint plots of the dihydrate.

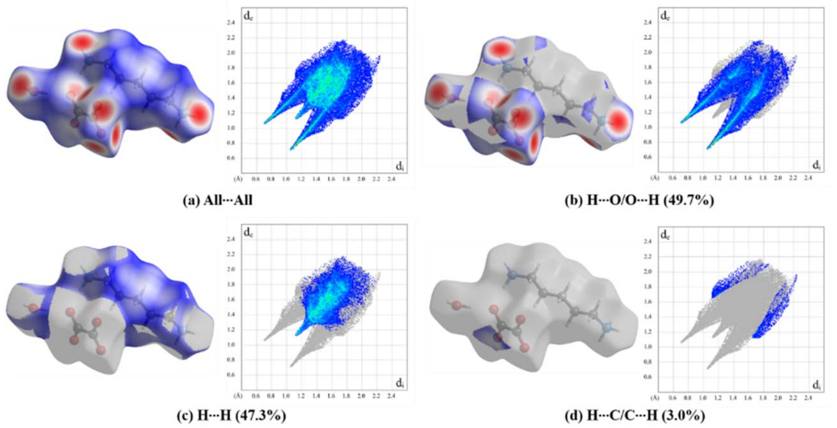

Figure S10 Hirshfeld surface and its corresponding fingerprint plots of the trihydrate.

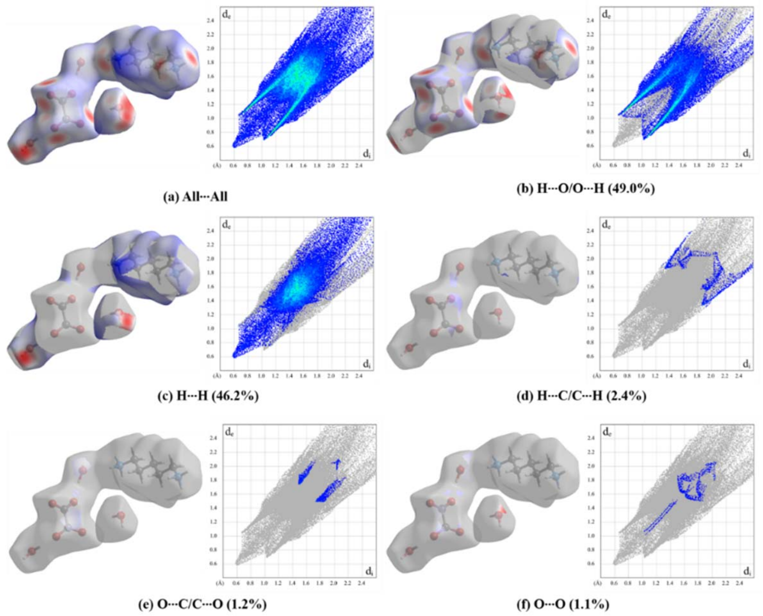

Figure S11 The Hirshfeld surface of two hydrates of PDA-OXA mapped over d_{norm} in the colour range -0.6615 to 1.1043 a.u. / -0.8808 to 2.2997 a.u. showing the hydrogen-bond interactions in the dihydrate (a-b) and trihydrate (c-d).

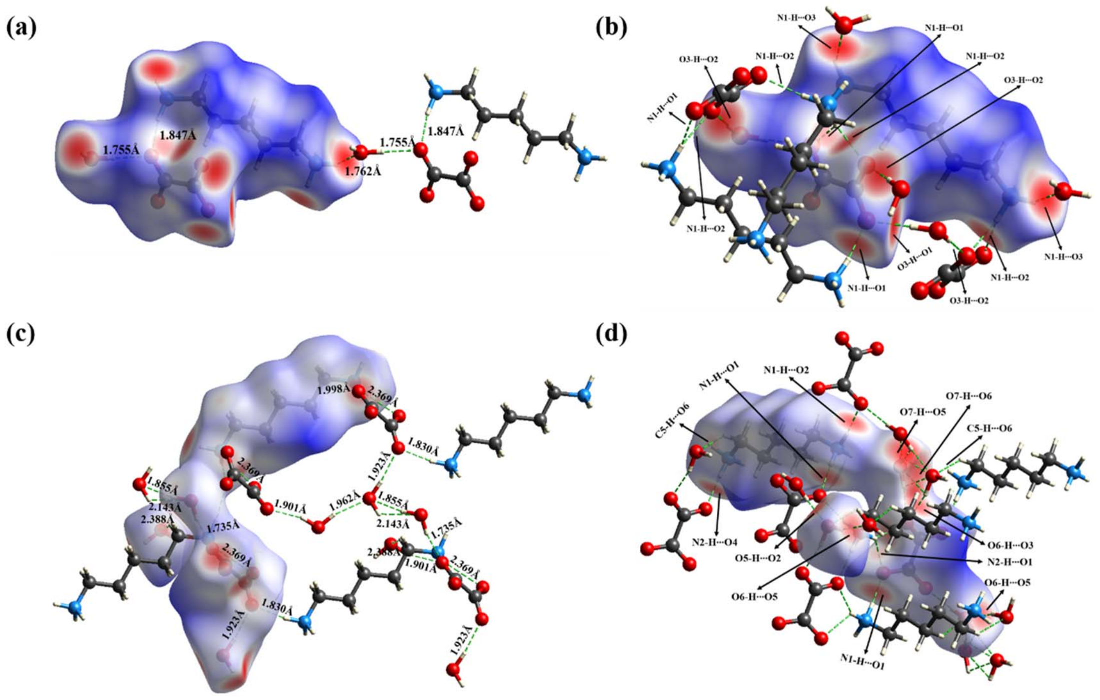

Table S3 Binding energy and lattice energy calculations for the PDA-OXA two hydrates.

	Dihydrate	Trihydrate
[a]E_{bind} (kcal/mol)	-26498.19	-7015.44
[b]E_{latt} (kcal/mol)	-280.98	-235.95

a: E_{bind} refers to the energy released when free atoms combine into crystal unit cells.

b: E_{latt} is the lattice energy, $E_{latt} = E_{bulk}/Z - E_A - E_B - E_C$[55], where E_{bulk} represents the total energy of the cell unit. E_A, E_B and E_C correspond to PDA, OXA and water molecules' relaxed energy, respectively, and Z is the number of heterodimers in the unit cell.

Figure S12VT-PXRD patterns of the dihydrate during heating, showing a new metastable crystal form appears after exceeding 120 °C.

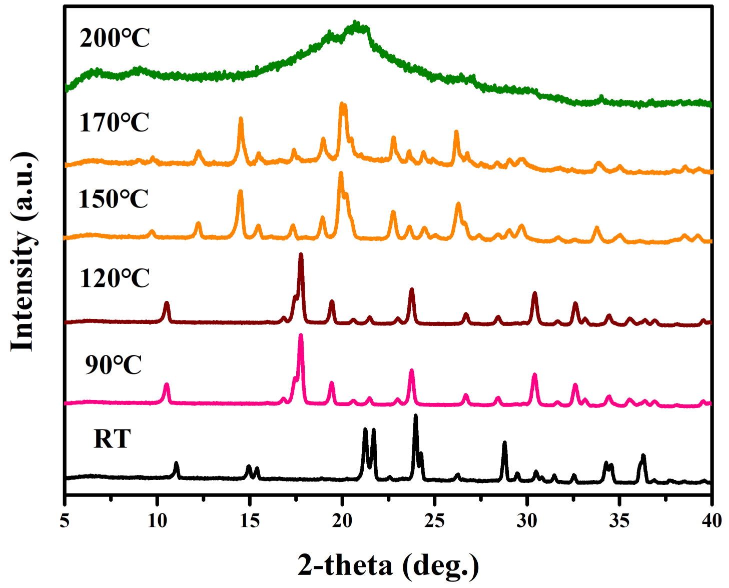

Figure S13 Hygroscopicity of two PDA-OXA crystal forms under the surrounding conditions with different relative humidity.

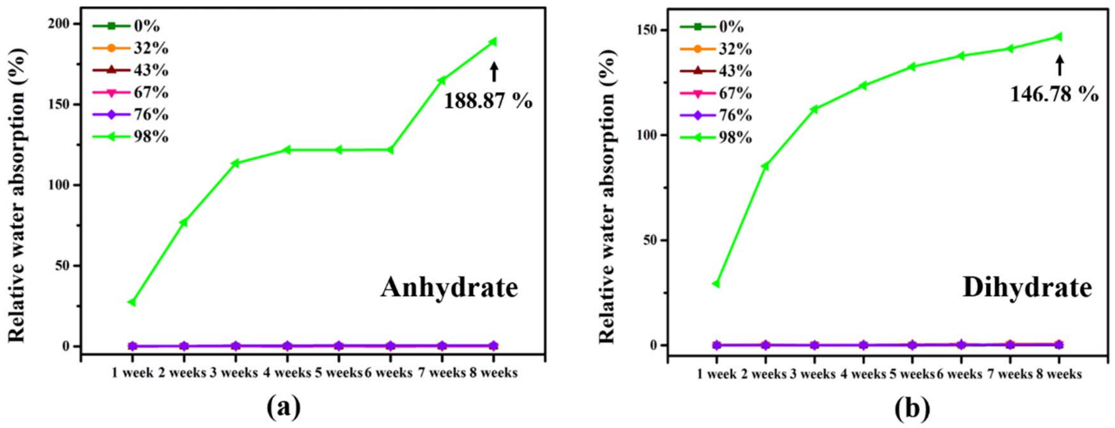

Figure S14 The macroscopic phenomenon of mass change of the anhydrous powders during the hygroscopic experiment was observed (RH=98%). (a): Initial sample; (b): 1 week later, the sample starts to absorb moisture; (c): 2 weeks later, almost all samples absorbed moisture and dissolved, accompanied by the formation of block dihydrate crystals; (d)-(f): the remaining anhydrate is completely dissolved, and the dihydrate crystals gradually grow up.

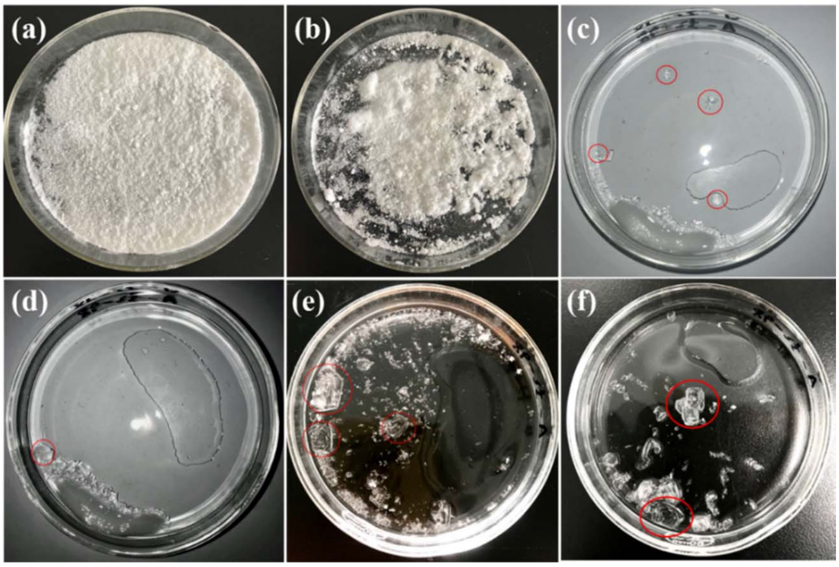

Figure S15 Stability experiment of PDA-OXA anhydrate under different water activity at 10℃-25℃.

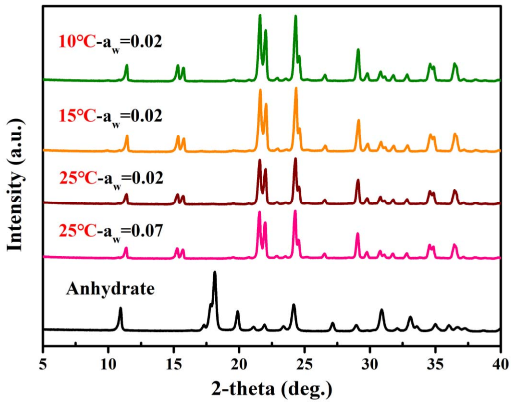

Figure S16 Detailed thermodynamic analysis of the obtained PA52 products by different polymerization methods. (a) is the TGA analysis while (b) is the DSC results.

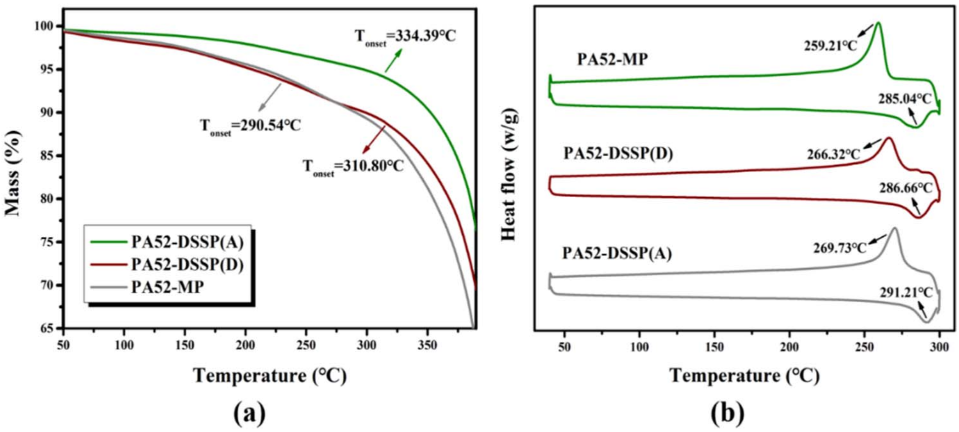

Supplement: Supplementary file 4 [file m-10-00052-sup4.pdf]
